# Exploring the Biomarkers of Sepsis-Associated Encephalopathy (SAE): Metabolomics Evidence from Gas Chromatography-Mass Spectrometry

**DOI:** 10.1155/2019/2612849

**Published:** 2019-11-12

**Authors:** Jing Zhu, Mu Zhang, Tingli Han, Hua Wu, Zhibo Xiao, Shihui Lin, Chuanjiang Wang, Fang Xu

**Affiliations:** ^1^Department of Critical Care Medicine, The First Affiliated Hospital of Chongqing Medical University, Chongqing, China; ^2^The Laboratory of Lipid & Glucose Metabolism, The First Affiliated Hospital of Chongqing Medical University, Chongqing, China; ^3^Liggins Institute, University of Auckland, Auckland, New Zealand; ^4^Center for Cognitive and Neurobiological Imaging, Stanford University, Stanford, CA, USA; ^5^Department of Radiology, The First Affiliated Hospital of Chongqing Medical University, Chongqing, China

## Abstract

**Background:**

Sepsis-associated encephalopathy (SAE) is a transient and reversible brain dysfunction, that occurs when the source of sepsis is located outside of the central nervous system; SAE affects nearly 30% of septic patients at admission and is a risk factor for mortality. In our study, we sought to determine whether metabolite changes in plasma could be a potential biomarker for the early diagnosis and/or the prediction of the prognosis of sepsis.

**Method:**

A total of 31 SAE patients and 28 healthy controls matched by age, gender, and body mass index (BMI) participated in our study. SAE patients were divided into four groups according to the Glasgow Coma Score (GCS). Plasma samples were collected and used to detect metabolism changes by gas chromatography-mass spectrometry (GC-MS). Analysis of variance was used to determine which metabolites significantly differed between the control and SAE groups.

**Results:**

We identified a total of 63 metabolites that showed significant differences among the SAE and control groups. In particular, the 4 common metabolites in the four groups were 4-hydroxyphenylacetic acid; carbostyril, 3-ethyl-4,7-dimethoxy (35.8%); malic acid peak 1; and oxalic acid. The concentration of 4-hydroxyphenylacetic acid in sepsis patients decreased with a decrease of the GCS.

**Conclusions:**

According to recent research on SAE, metabolic disturbances in tissue and cells may be the main pathophysiology of this condition. In our study, we found a correlation between the concentration of 4-hydroxyphenylacetic acid and the severity of consciousness disorders. We suggest that 4-hydroxyphenylacetic acid may be a potential biomarker for SAE and useful in predicting patient prognosis.

## 1. Introduction

Sepsis is the second most common cause of mortality (representing the causative factor in more than 25% of cases), with increasing incidence observed in intensive care unit (ICU) patients [1, 2]. Of the types of tissue and organ dysfunction associated with sepsis, sepsis-associated encephalopathy (SAE) is a particularly important kind. SAE is a transient and reversible brain dysfunction condition that occurs when the source of sepsis is located outside of the central nervous system [3]. Approximately one-third of septic patients are afflicted with SAE, which represents a risk factor for long-term disability and mortality (16%–65%).

The quick Sepsis-Related Organ Failure Assessment (qSOFA) score is a good bedside tool to identify patients at risk of death and when predicting prognosis [4]. However, to date, there are no studies in existence that confirm that qSOFA is an effective indicator for assessing the severity of SAE. Still, it is a possibility that this scoring system has a certain relationship with the early identification and diagnosis of SAE. Currently, electroencephalography (EEG) and brain magnetic resonance imaging (MRI) are auxiliary tests typically used to evaluate electrical activity or focal injuries of the brain. However, as a common method for detecting neurological diseases, EEG is not always needed for the diagnosis of acute brain dysfunction or delirium in septic patients [5] and is more suitable for use in cases of SAE accompanied by seizures (present in up to 15% of cases) [6]. In contrast, MRI can reveal the brain injury during SAE, but it is difficult to define whether the injury is in its early stage. At this stage, it is not common to screen for SAE by using neuroelectrophysiological or imaging methods. When we focused on SAE from another angle, we found that the neuropathological hallmark of SAE is diffuse neuroinflammation and microcirculatory alterations [7]. Furthermore, the impaired vascular function and imbalance of neurotransmitters leading to excitotoxicity contribute significantly to brain dysfunction [4]. Sepsis is defined as a life-threatening organ dysfunction caused by a dysregulated host response to infection [8]. Currently, there is no hallmark biomarker for SAE though elevated plasma levels of calcium-binding protein B (S100*β*) or neuron-specific enolase (NSE) have been reported as possibilities [9]. Thus, this study sought to explore potential biomarkers for diagnosing or predicting the probability or duration of SAE. We used gas chromatography-mass spectrometry (GC-MS) to detect clinically paired study samples to find differences in the plasma metabolites of SAE patients.

## 2. Materials and Methods

### 2.1. Study Design

**Figure 1:**
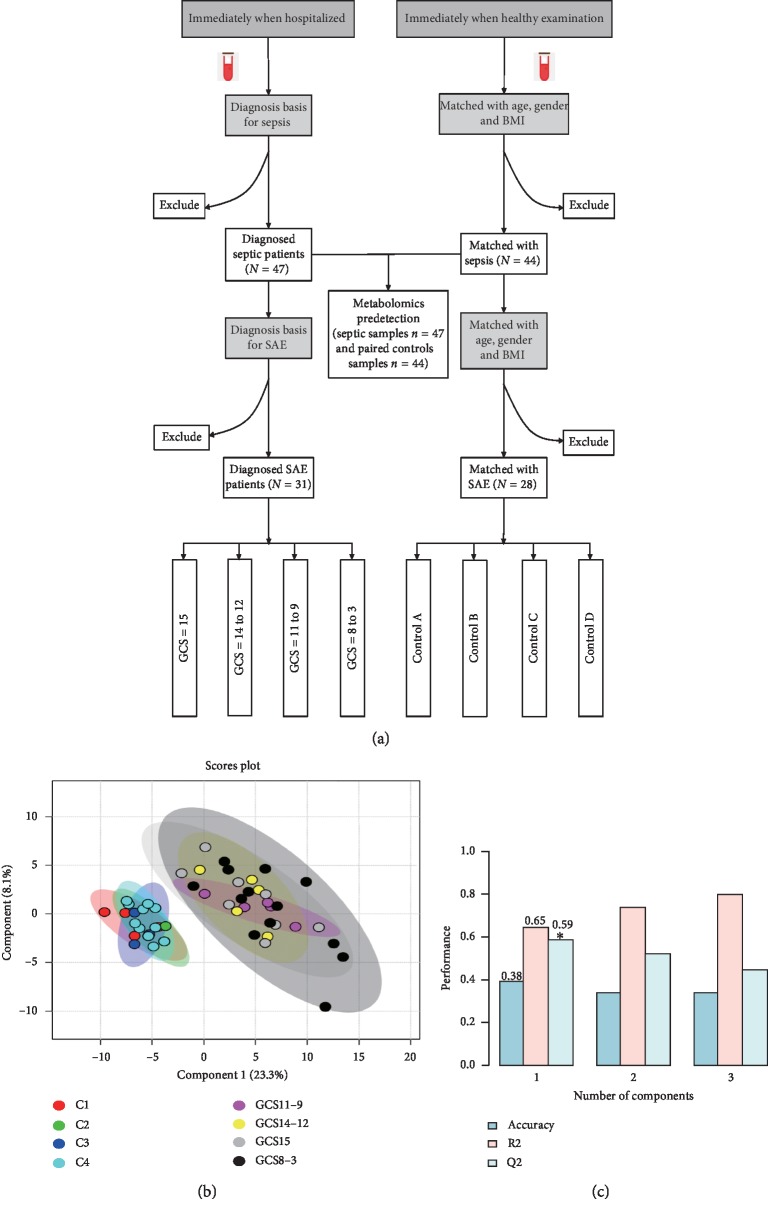
(a) The flow chart of our study. 47 septic patients and 44 paired healthy controls were in our study for metabolomic predetecting (total samples *n* = 91). Then, 31 out of 47 patients were diagnosed as SAE. According to the GCS, they were divided into 4 groups. (b) The PLS-DA of our study. (c) The leave-one-out cross-validation of our study.

This study was approved by the ethics committee of the First Affiliated Hospital of Chongqing Medical University in Chongqing. Study participants were identified from the ICU of the hospital between November 2015 and September 2016. The patients or their family members were fully informed about the study, and we obtained their written consent. 47 septic patients were recruited to our study, and 47 blood samples were collected (donor number = 47 and sample number = 47). As for healthy controls, 44 volunteers (which were from the Healthy Examination Center) provide blood samples to pair with septic patients (matched with age, gender, and body mass index). Within one hour of admitting by hospital, plasma samples were collected and the Glasgow Coma Score (GCS) which is commonly used to evaluate for mental status assessment in acute care was assessed immediately. Therefore, in the first step of our study, 47 septic samples (*n* = 47) and their healthy control samples (*n* = 44) were preformed metabolomic testing (total sample *n* = 91). Next, one of the auxiliary inspection methods for SAE, including the electroencephalography (EEG), cranial magnetic resonance imaging (MRI), cranial computed tomography (CT), and transcranial Doppler (PCD), was used to screen SAE patients from septic patients. Finally, 31 out of 47 septic patients were diagnosed as SAE. According to the GCS, SAE patients were divided into the following four groups: GCS score = 15 points, GCS score = 14 points to 12 points, GCS score = 11 points to 9 points, and GCS score = 8 points to 3 points, respectively. Finally, 28 of 44 paired healthy controls corresponded to SAE patients with different GCS scores (Control A/GCS, 15; Control B/GCS, 14–12; Control C/GCS, 11–8; and Control D/GCS ≤8) (Figure 1(a)).

### 2.2. Sample Preparation and Labeling with GC-MS

To deproteinise the plasma samples, 800 *μ*l of prechilled methanol (containing internal standard 2,3-3-3-d4-alanine, 0.3 *μ*mol) was mixed into the 200 *µ*l of plasma aliquots and subsequently frozen at −20°C for 30 min. The precipitated proteins were eliminated by centrifugation at 12,000 rpm for 15 min. The remaining supernatants were then dehydrated in a SpeedVac (Labconco, USA) at room temperature for 7 hours and stored in −80°C prior to chemical derivatisation.

### 2.3. GC-MS Analysis

The dried samples were chemically derivatized via the methyl chloroformate (MCF) method in accordance with the protocol published by Smart et al. [10] The dried extracts were resuspended in the order of following solutions: 200 *μ*L of sodium hydroxide (1 M), 167 *μ*L of methanol, and 34 *μ*L of pyridine. To commence the MCF derivatization, 20 *μ*L of MCF was added and mixed for 30 seconds twice. To stop derivatization, 400 *μ*L chloroform and 400 *μ*L of bicarbonate (50 mM) were added and mixed 10 s each time. The mixture was then centrifuged for 2 min at 2000 rpm to isolate the organic layer from the aqueous layer. Lastly, the aqueous layer was removed and added anhydrous sodium sulphate to eliminate the remaining water. The organic layer containing derivatized metabolites was transferred into GC vials readily for GC-MS analysis. For GC-MS, a ZB-1701 gas-phase capillary column (30 m × 320 *μ*m × 0.25 *μ*m; Phenomenex, Torrance, CA, USA), inlet for a splitter/splitless inlet (Agilent Technologies, Santa Clara, CA, USA) was used, with an inlet temperature of 250°C, a flame ionization detector (FID) detector temperature of 250°C, a nitrogen flow rate of 40 mL/min, a hydrogen flow rate of 40 mL/min, an air flow rate of 450 mL/min, a split ratio of 20 : 1, and a sample injection volume of 1 *μ*l. In addition, the temperature protocol was as follows: an initial temperature of 80°C, with an increase from 25°C/min to 200°C, and then with an increase from 3°C/min to 215°C. Finally, the metabolites were analyzed at an increase from 2°C/min to 230°C.

### 2.4. Data Normalization and Statistical Analysis

AMDIS (VS2.71, from OpenChrom) software was used to deconvolute the GC-MS spectra, and the mass spectrometry library of the MCF-identified compounds was derived from Smart et al. [10] (from SHIMADZU, Japan). The metabolites were labeled by MCF-treated derivatives of the mass spectrum and the corresponding mass retention time. The relative intensities of the identified metabolites were calculated by the XCMS-based R-script, and the selected base ion of the reference ion was determined to be within the appropriate retention time. Metabolite abundance was normalized by the internal standard number of each sample, and the batch error was removed by the median number. Identified metabolites were from the MS library, while metabolites not recognized in the MS library could be found in NIST, and the degree of matching with the compound in the NIST was marked in the form of *n*% after the name of the metabolite. After sorting out the metabolites, we evaluated samples by GC-MS and *t*-test, and the error rate was used to predetermine metabolic changes between the sepsis and control groups. After diagnosed as SAE, they were grouped according to GCS, and analysis of variance (ANOVA) was used to filter out metabolites with significant differences. The resulting data were drawn by the ggplot2 R programs (R Development Core Team, New Zealand).

## 3. Results

### 3.1. Analysis of SAE Patients Based on the Disturbance of Consciousness Aggravation with GCS

**Figure 2:**
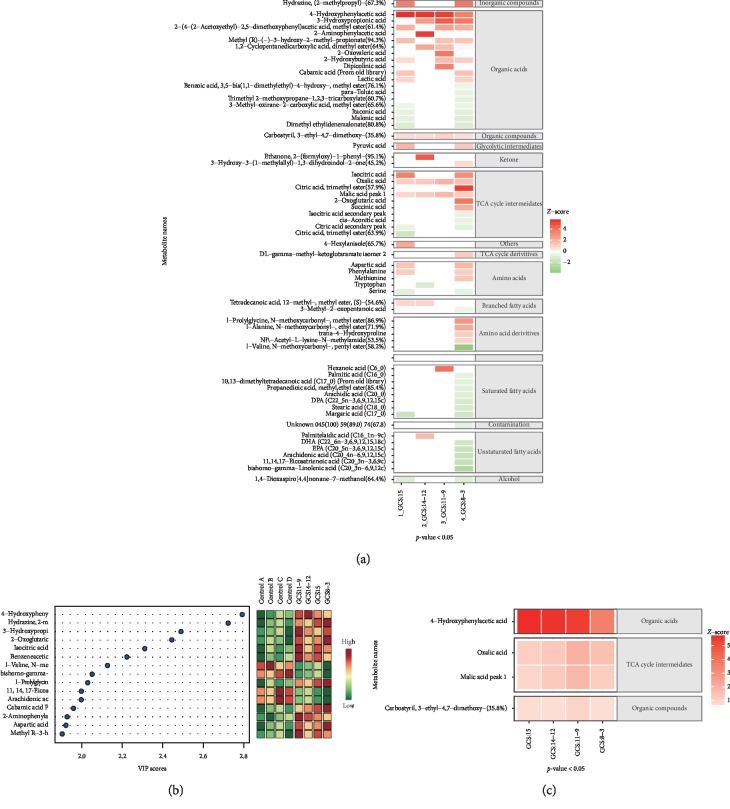
(a) Metabolites' concentration gradient map after the ANOVA test. ^*∗*^*p* < 0.05, comparison of each GCS degrees of SAE groups and control groups. The name of metabolites is showing at the left, and the degree of matching with the compound in the NIST was marked in the form of *n*% after the name of the metabolites. The classification of the metabolites is on the right. (b) The VIP score analysis of our study shows out the best 10 metabolites, showing changes in different groups. (c) The heatmap of 4 common metabolites.

The basic information for the SAE patient samples and paired healthy controls is listed in Table 1 and is grouped according to GCS. PLS-DA and leave-one-out cross-validation are shown in Figures 1(b) and 1(c). Before the statistical analysis, we calculated the VIP scores to indicate the best 10 metabolites in our study (Figure 1(b)). After ANOVA was performed for the GCS and control groups, 63 metabolites were identified with statistical significance (*p* < 0.05) (Figure 2(a)).

When considering the GCS = 15 points and matched control patients, 25 metabolites demonstrated statistical significance (^*∗*^*p* < 0.05). We also found that 3 amino acids were changed in the GCS = 15 points group of SAE, including aspartic acid, phenylalanine, and serine. Among them, aspartic acid and phenylalanine were increased, but serine was decreased. Furthermore, of the 5 tricarboxylic acid (TCA) cycle intermediates, 40% (2/5) of them were decreased and 60% (3/5) were increased in the GCS = 15 points group. Meanwhile, of the 2 fatty acids with tetradecanoic acid (12-methyl-, methyl ester, and (*S*)-), one of the branch fatty acids was increased, but margaric acid, a saturated fatty acid, was decreased. Furthermore, pyruvic acid, an important metabolite of the glycolytic intermediates group, was found to be increased in the GCS = 15 points group. Ten organic acids were found, with 60% (6/10) of them being increased, including 4-hydroxyphenylacetic acid (4-HPA), 2-hydroxybutyric acid, methyl (*R*)-(−)-3-hydroxy-2-methyl-propionate, 3-methyl-oxirane-2-carboxylic acid (methyl), itaconic acid, and lactic acid. The others, [2-(4-(2-acetoxyethyl)-2,5-dimethoxyphenyl)acetic acid (methyl ester), 2-hydroxybutyric acid, carbamic acid, and malonic acid, were decreased.

In the GCS = 14 points to 12 points group, 11 metabolites demonstrated statistical significance (^*∗*^*p* < 0.05). The amino acid tryptophan was increased in the GCS = 14 points to 12 points group, and 2 TCA cycle intermediates (malic acid and oxalic acid) were similarly increased. In addition, 4 organic acids (i.e., 4-HPA, 1,2-cyclopentanedicarboxylic acid (dimethyl), 3-hydroxypropionic acid, and 2-aminophenylacetic acid) were all found to be increased. Two fatty acids, palmitelaidic acid and an unsaturated fatty acid, were decreased, while tetradecanoic acid (12-methyl-, methyl ester, and (*S*)-), one of the branched fatty acids, was increased.

In the GCS = 11 points to 9 points group, 12 metabolites showed statistical significance (^*∗*^*p* < 0.05). All (12/12) metabolites were increased in the GCS = 15 points group, including 8 organic acids (4-HPA, (2-(4-(2-acetoxyethyl)-2,5-dimethoxyphenyl)acetic acid (methyl ester), 2-hydroxybutyric acid, methyl (*R*)-(−)-3-hydroxy-2-methyl-propionate, 1,2-cyclopentanedicarboxylic acid (dimethyl), 3-hydroxypropionic acid, 2-oxovaleric acid, and dipicolinic acid), 2 TCA cycle intermediates (malic acid and oxalic acid), and one saturated fatty acid (hexanoic acid).

In the GCS = 8 points to 3 points group, 51 metabolites demonstrated statistical significance (^*∗*^*p* < 0.05). Here, we found 9 amino acids. 5 of them were amino acid derivatives (i.e., *l*-alanine (*N*-methoxycarbonyl-, ethyl ester), *l*-prolylglycine (*N*-methoxycarbonyl-, methyl ester), *l*-valine (*N*-methoxycarbonyl-, pentyl ester), *N*-Acetyl-*L*-lysine-*N*-methylamide, and *trans*-4-hydroxyproline) and were increased in the GCS = 8 points to 3 points group, while the other 4 were amino acids. Half (2/4) of the latter group (phenylalanine and serine) were decreased, while the other 2 (aspartic acid and methionine) were increased. Thirteen fatty acids including 5 unsaturated fatty acids, 7 saturated fatty acids, and one branched fatty acid were found. The branched fatty acid 3-methyl-2-oxopentanoic acid and 28.6% (2/7) of saturated fatty acids (palmitic acid and stearic acid) were increased, while 71.4% (5/7) of saturated fatty acids (margaric acid, arachidic acid, docosapentaenoic acid (DPA), 10,13-dimethyltetradecanoic acid, propanedioic acid (methyl, ethyl ester), propanedioic acid (methyl, ethyl ester)) were decreased. In addition, 40% (2/5) of the unsaturated fatty acids (bishomo-gamma-linolenic acid and docosahexaenoic acid (DHA)) were increased, but others including 11,14,17-eicosatrienoic acid, arachidonic acid, and eicosapentaenoic acid (EPA) were decreased. We also found that the glycolytic intermediate pyruvic acid was increased. Fourteen organic acids were found, with 50% of them being decreased (i.e., 2-(4-(2-acetoxyethyl)-2,5-dimethoxyphenyl)acetic acid (methyl ester), ethyl (R)-(−)-3-hydroxy-2-methyl-propionate, 3-methyl-oxirane-2-carboxylic acid (methyl ester), itaconic acid, malonic acid, 3-hydroxypropionic acid, and trimethyl 2-methoxypropane-1,2,3-tricarboxylate) and 50% of them being increased (i.e., 4-HPA, 2-hydroxybutyric acid, carbamic acid, dimethyl ethylidenemalonate, lactic acid, benzoic acid, 3,5-bis(1,1-dimethylethyl)-4-hydroxy-(methyl ester), and *para*-toluic acid).

According to the results obtained in comparison with those of samples and controls, 4 metabolites had common statistical significance. All 4 of these metabolites were increased in the study samples (Figure 2(c)). More importantly, we found that 4-HPA was high in SAE patients, and the level of 4-HPA in the GCS = 15 points, GCS = 14 points to 12 points, and GCS = 11 points to 9 points groups stayed high but was decreased in the GCS = 8 points to 3 points group compared with other 3 groups. After common metabolites were detected among the 4 groups, correlation analyses between 4-HPA and sepsis-related scores (e.g., acute physiology and chronic health evaluation (APACHE) II, APACH III, Sequential Organ Failure Assessment (SOFA), and quick Sequential Organ Failure Assessment (qSOFA)) were calculated. With statistically significant *p* values but poor *r* values and 95% confidence intervals, the results showed statistical differences with APACHE II, APACHE III, and qSOFA. Combined with the slope and 95% CI, the results do not show the clinical perspective more aptly, instead revealed more significant differences ([Supplementary-material supplementary-material-1]). However, only 4-HPA maintained high levels and decreased gradually in GCS 15, GCS 14-12, and GCS 11-9. In particular, after GCS≤8, the difference of 4-HPA was significant (*p*=0.012), compared with the above three groups. In addition, there was a nonlinear relationship (*R*^2^ = 0.04177) between 4-HPA and GCS.

### 3.2. Analysis of Predicting Metabolic Pathways of SAE as the Disturbance of Consciousness Aggravation with GCS

**Figure 3:**
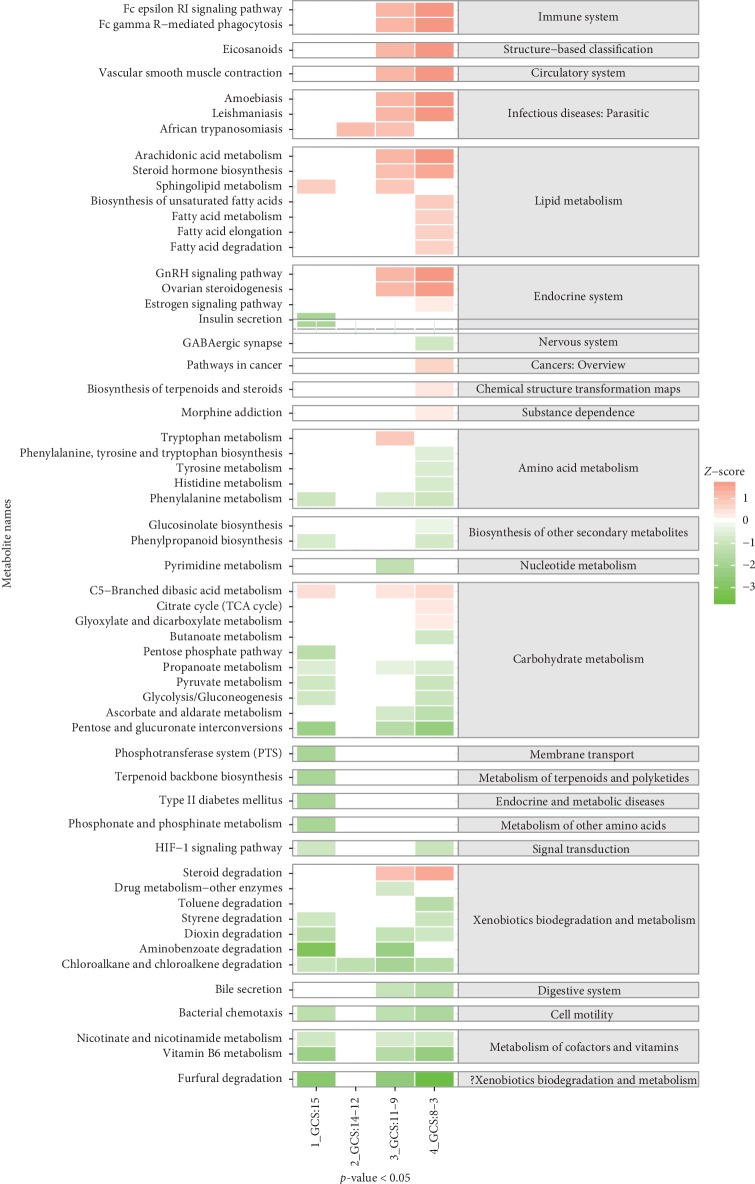
Concentration gradient map of metabolite pathways after the *T* test. ^*∗*^*p* < 0.05, comparison of each SAE groups and control groups. The classifications of the pathways are on the right.

While recognizing the linked metabolic pathways, the ANOVA test was used to extract significant pathways from them. There were 58 metabolic pathways identified as part of our study (Figure 3). Comparing the GCS = 15 points patients, 23 metabolic pathways were found and 2 amino acid-connected pathways (phenylalanine metabolism and phosphonate and phosphinate metabolism) were decreased in the GCS = 15 points group. Meanwhile, only a single lipid metabolism (sphingolipid metabolism) was found to be increased. Seven metabolic pathways associated with energy metabolism were also identified, and only C5-branched dibasic acid metabolism was increased. Importantly, the signal transduction of the hypoxia-inducible factor-1 (HIF-1) signaling pathway, which is associated with sepsis, was found to be decreased. Comparing the GCS = 14 points and 12 points patients, only two metabolic pathways were found (Figure 3).Comparing the GCS 11 points with 9 points patients, 30 metabolic pathways were found. The results specifically showed that phenylalanine metabolism decreased and tryptophan metabolism increased in the samples. The metabolism of the immune system (Fc epsilon RI signaling pathway and Fc gamma R-mediated phagocytosis) and 3 aspects of the lipid system (sphingolipid metabolism, arachidonic acid metabolism, and steroid hormone biosynthesis) were all increased. Forty-six metabolic pathways had statistical significance in the GCS = 8 points to 3 points group.

## 4. Discussion

The current understanding of the pathogenesis of SAE is multifactorial and unclear, including aspects such as microcirculatory dysfunction and occurring under perfusion involving damage, ischemia, and cellular death, and in a systemic inflammatory state involving leukocytes or/and microglial activation, lysosomal exocytosis, cytokine release, and free-radical generation, as well as in conjunction with an energy metabolism disorder involving mitochondrial dysfunction [3]. With the progress of sepsis, inadequate cerebral perfusion caused by the dysfunction of cerebral autoregulation, apoptosis, impaired microcirculation, and neurotoxic substance neurotransmitters may eventually lead to SAE. Host cells play various biological roles, and metabolism analysis could show the abnormalities in the tissue and cells of SAE, thus contributing to discovering new indicators for early diagnosis or therapy. In our clinical pairing study, we found that there were 63 different metabolites (*p* < 0.05) and 58 different metabolic pathways (from Kyoto Encyclopedia of Genes and Genomes (KEGG) data) between the SAE group and control groups (*p* < 0.05). These findings show that there are many types of differential metabolites accompanied by SAE, which could point to a number of predictable metabolic pathways. However, identifying commonalities among so many different metabolites and metabolic pathways still requires a process of clinical thinking.

We used the deterioration of the consciousness barrier of SAE patients as an entry point. This is often the primary concern of ICU doctors in making the judgments about SAE. Considering the results we found, as the conscious disorder of sepsis encephalopathy worsened, the common metabolites changed to reveal key clues in the appearance and progress of SAE. Four metabolites including 4-HPA, carbostyril (3-ethyl-4,7-dimethoxy-), malic acid peak 1, and oxalic acid were meaningful under different GCS conditions between the SAE and control groups. Based on the composition of the score, 4-HPA is a GCS-related indicator, and the correlation with the overall patient severity requires additional research evidence. The greater significance of 4-HPA screening is that the changes may be used as an indicator for dynamic assessment in SAE.

Some studies have shown that 4-HPA markedly suppressed the levels of cytochrome P450 2E1 (CYP2E1) expression and enhanced antioxidant enzyme levels. It has been suggested that 4-HPA has a potential antioxidative effect [11]. As an antioxidative, it is known to attenuate hypoxia, inflammation, vascular leak, and edema and to decrease HIF-1*α* protein levels. HIF-1, composed of 2 submits including HIF-1*α* and HIF-1*β*, is the key transcription factor that mediates adaptive responses to changes in tissue oxygenation. The HIF-1*α* protein is kept at a low or undetectable level by continuous HIF-prolyl hydroxylase domain enzyme-mediated degradation, which is suppressed by hypoxia [12, 13]. The HIF-1 plays a crucial role in sepsis. The expression level if HIF-1 can be induced in response to reduced oxygen availability and other stimulants (such as nitric oxide (NO) or various growth factors) in metabolic processes [14]. Trained monocytes display high glucose consumption and lactate in sepsis [15]. In addition mTOR induction of glycolysis is mediated through activation of HIF-1*α*, which was influenced by the genetic polymorphism of mTOR and glycolytic [16]. Another study showed the reason for the decline in HIF-1*α* levels. With the aggravating condition of sepsis, on the one hand, the HIF = 1 target gene expression was decreased; on the other hand, the HIF-1*α* mRNA expression was decreased. The reduced source of HIF-1*α* may cause the decreased HIF-1*α* in plasma of septic patients [17]. Therefore, HIF-1*α* remains in a dynamic status; the HIF-1 signaling pathway could be increased, decreased, or unchanged in patients with sepsis. During sepsis, cells are in a state of hypoxia and potentially show high expression levels of HIF-1*α* protein. A separate study showed that 4-HPA reduced inflammatory cytokine levels through suppressing hypertonicity- and hypoxia-induced HIF-1*α* in macrophages [12]. This funding suggests the reason that the abnormally elevated 4-HPA levels reduced in serum samples with poor GCS may be involved in reductions in inflammation and hypoxia after high HIF-1 expression.

Oxidative stress, the unbalancing of oxidants and antioxidants in the body, is believed to be a mechanism of sepsis associated with organ dysfunction [18, 19]. Peroxynitrite, the product of the reaction of nitric oxide (NO) and superoxide (O_2_−), –is a key harmful molecule that may be detoxified by reacting with tyrosine [20]. Some researchers have reported that neutrophil-derived myeloperoxidase uses chloride, and halogenated tyrosine residues are commonly used to quantify leukocyte-mediated damage in diseased tissues. In a recent investigation, 4-HPA was confirmed to participate in the intermediate step of tyrosine degradation [20]. It is known that chlorotyrosine is metabolized to 3-chloro-4-hydroxyphenylacetic acid (chloro-HPA) and that 4-HPA is metabolized by urine. Furthermore, monooxygenases are enzymes that catalyze the initial reactions of aerobic catabolic pathways by incorporating an oxygen atom in various aromatic compounds. Notably, 4-hydroxyphenylacetate-3-hydroxylase (HPAH), as one of the monooxygenases that has been found in various bacteria, can catalyze the hydroxylation of 4-HPA to 3,4-dihydroxyphenylacetic acid, which is the initial step in the aerobic degradation pathway of 4-HPA [20, 21]. At present, major urinary metabolites of chlorotyrosine and nitrotyrosine have been identified [22–24], and their measurement has been employed for the assessment of systemic inflammation in vivo [25, 26]. Because oxidized proteins undergo proteolysis, the resulting free halogenated amino acids are metabolized and excreted in the urine. Importantly, the metabolite 4-HPA could be a biomarker for quantifying leukocyte-mediated damage [27]. Thus, elevated levels of 4-HPA in the serum samples of SAE patients may be associated with infection directionality [28]. This represents a possible direction for further exploration.

Another interesting inference about the role of 4-HPA comes from the following evidence. There is a consensus that compounds acting as modulators of brain function subsequently need to cross the blood-brain barrier (BBB) to reach the central nervous system though a previous study about the value of 4-HPA as a biomarker in depression and anxiety showed an interesting finding: the researchers found a decrease in 4-HPA levels in both plasma and cerebrospinal fluid during depressive or anxiety episodes [29]. Thus, according to *in vitro* BBB research in rats and humans, 4-HPA has poor BBB permeability [30]. Therefore, one question to consider is how do we look upon our present results that suggest that a high level of 4-HPA in plasma may indicate differences in stress-induced changes after SAE between the patients with GCS = 15 points and GCS = 14 to 3 points. In fact, 4-HPA, as one of the major metabolites in polyphenols, is derived from polyphenols by colonic microflora and could be absorbed into the systemic circulation. We know that there are pathways of bidirectional communication between the brain and the gut, including the autonomic nervous system (ANS), enteric nervous system (ENS), neuroendocrine system, and immune system. During sepsis, the above communication route will afford bacteria an opportunity to translocate across the intestinal mucosa and directly access both immune and neuronal cells of the ENS, as a necessary adaptive response of microbiota to the stress-induced changes in inflammation [28]. Therefore, during the development of SAE, 4-HPA potentially plays an indirect stimulating role via involvement of the gut-brain axis. It is also possible that the role of 4-HPA is through the other metabolites of colonic microflora, such as *p*-hydroxybenzoic acid, phenylacetic acid, and 4-methoxyphenylacetic acid [30]. Interestingly, a recent study showed that 3,4-dihydroxyphenylacetic acid is also one of the metabolites of dopamine (DA); furthermore, it can cross the BBB and the levels of 3,4-dihydroxyphenylacetic acid could change in the brain in a regional- and dose-related manner [31]. Some research has shown that the presence of pathogenic bacteria in the gastrointestinal tract (GI tract), in the absence of a systemic immune response, can increase anxiety-like behavior [32, 33]. This can be used as indirect evidence to explain why the level of 4-HPA is decreased as the disturbance of consciousness is aggravated.

In summary, the metabolic disorder of cells may be an important part of the development of SAE. However, at this time, the mechanism of 4-HPA in the central nervous system is not clear. Some studies show that behavioral changes are induced by i.p. administration of 4-HPA [34]. In addition, 4-HPA could be absorbed from the gut lumen via an active transporter, the monocarboxylic acid transporter [35], to enter the systemic circulation, which is also expressed on the luminal membrane of endothelial cells at the BBB [36]. This may be connected with the level of 4-HPA in the plasma and changes in mental behavior. The metabolites we found in this study show the abnormal metabolic traces associated with anti-inflammatory processes, antioxidation, and behaviors of depression and anxiety in SAE. The finding that 4-HPA is accompanied by a change in the severity of consciousness disorders is an interesting result and may indicate that 4-HPA could be a potential biomarker for the early identification of or in assessing the severity of SAE.

In metabolic analysis, dynamic changes in 4-HPA may serve as biomarkers for early diagnosis and dynamic follow-up of SAE. Our study used metabolomics analysis, based on a 1 : 1 paired study between sepsis and healthy control, to confirm SAE. This research strategy is more common in metabolomics analysis, but there is some difference in the pairing of SAE and sepsis, which is more closely related to clinical practice. Therefore, the changes in 4-HPA seemed to be of exploratory significance. But at least, our research suggested that the use of plasma metabolomics analysis is an option for finding biomarkers for SAE. However, due to the limitations of clinical conditions, a large sample pairing study should be conducted.

## Figures and Tables

**Table 1:** Basic formation of groups.

Groups	Numbers	Age	BMI	Gender	Apache II	Apache III	qSOFA	SOFA
GCS 15	8	67.25 ± 12.44	20.90 ± 2.96	5 : 3	19.5 ± 6.43	59.12 ± 15.51	1.37 ± 0.51	12 ± 4.53
GCS 14∼12	5	72.80 ± 7.88	18.52 ± 1.64	4 : 1	29.2 ± 4.65	82.2 ± 27.52	1.8 ± 0.44	13.6 ± 4.97
GCS 11∼9	5	68.80 ± 19.01	21.80 ± 3.36	2 : 3	25.8 ± 11.81	75.2 ± 25.60	1.8 ± 0.83	13.8 ± 3.63
GCS 8∼3	13	69.69 ± 17.4852	24.08 ± 5.68	8 : 5	27 ± 9.44	76.61 ± 26.65	2.15 ± 0.80	13.53 ± 4.96
Control A	8	66.37 ± 12.56	21.10 ± 2.86	6 : 2	—	—	—	—
Control B	5	73.00 ± 7.90	19.76 ± 2.10	4 : 1	—	—	—	—
Control C	4	66.5 ± 17.40	20.52 ± 3.88	2 : 2	—	—	—	—
Control D	11	69.90 ± 16.98	21.87 ± 3.34	7 : 4	—	—	—	—

Control A is linked with the control of GCS 15. Control B is linked with the control of GCS 14∼12. Control C is linked with the control of GCS 11∼9. Control D is linked with the control of GCS 8∼3. The numbers were recorded as mean ± SD.

## Data Availability

The data used to support the findings of this study are available from the corresponding author upon request.

## Supplementary Materials

Supplementary Materials Figure legend Supplement 1 A. The correlation analysis between 4-HPA and APACHE II. B. The correlation analysis between 4-HPA and APACHE III. C. The correlation analysis between 4-HPA and SOFA. D. The correlation analysis between 4-HPA and qSOFA. We found the *p* values in correlation analysis of 4-HPA with APACHE II, APACE III, and qSOFA. *P* values are less than 0.05, but the *r* and 95% CI is poor. It showed the clinical perspective more aptly, but more statistical differences. Figure legend Supplement 2 A. During the progress of sepsis, 4-HPA may have a potential antioxidative effect by cytochrome P450 2E1 to elevate the expression of HIF-1 pathways. B. Some research studies showed out that 4-HPA is one of the intermediate steps of chlorotyrosine, it can catalyze the hydroxylation of 4-HPA to 3,4-dihydroxyphenylacetic acid by 4-hydroxyphenylacetate3-hydroxylase (HPAH) which is one of the monooxygenases found in various bacteria; and it could be tested in urine to quantify leukocyte-mediated damage in diseased tissues. C. Because of the disorders of bidirectional communication between the brain and the gut, there are two hypotheses: one is that the gut-brain axis could offer the chances of bacteria to translocate across the intestinal mucosa and act on immune cells and neuronal cells, and it could lead to behavior changes. Two is that 4-HPA can be absorbed through gut-brain axis, and the other metabolites of 4-HPA such as dopamine (DA) could be the participants of the factors of behavior changes.
