# Supplementary material for: Exploring the Biomarkers of Sepsis-Associated Encephalopathy (SAE): Metabolomics Evidence from Gas Chromatography-Mass Spectrometry
Source: Biomed Res Int. 2019 Nov 12;2019:2612849. doi: 10.1155/2019/2612849 (PMC6875220; doi:10.1155/2019/2612849)
Supplement: Supplementary Materials — Figure legend Supplement 1 A. The correlation analysis between 4-HPA and APACHE II. B. The correlation analysis between 4-HPA and APACHE III. C. The correlation analysis between 4-HPA and SOFA. D. The correlation analysis between 4-HPA and qSOFA. We found the p values in correlation analysis of 4-HPA with APACHE II, APACE III, and qSOFA. P values are less than 0.05, but the r and 95% CI is poor. It showed the clinical perspective more aptly, but more statistical differences. Figure legend Supplement 2 A. During the progress of sepsis, 4-HPA may have a potential antioxidative effect by cytochrome P450 2E1 to elevate the expression of HIF-1 pathways. B. Some research studies showed out that 4-HPA is one of the intermediate steps of chlorotyrosine, it can catalyze the hydroxylation of 4-HPA to 3,4-dihydroxyphenylacetic acid by 4-hydroxyphenylacetate3-hydroxylase (HPAH) which is one of the monooxygenases found in various bacteria; and it could be tested in urine to quantify leukocyte-mediated damage in diseased tissues. C. Because of the disorders of bidirectional communication between the brain and the gut, there are two hypotheses: one is that the gut-brain axis could offer the chances of bacteria to translocate across the intestinal mucosa and act on immune cells and neuronal cells, and it could lead to behavior changes. Two is that 4-HPA can be absorbed through gut-brain axis, and the other metabolites of 4-HPA such as dopamine (DA) could be the participants of the factors of behavior changes. [file 2612849.f1.pdf]

Supplement 1

A. The correlation analysis between 4-HPA and APACHE II.

B. The correlation analysis between 4-HPA and APACHE III.

C. The correlation analysis between 4-HPA and SOFA.

D. The correlation analysis between 4-HPA and qSOFA.

We found the p-values in correlation analysis of 4-HPA with APACHE II, APACE III and qSOFA. P values are less than 0.05, but the r and 95%CI is poor. It showed the clinical perspective more aptly, but more statistical differences.

Supplement 2

A. During the progress of sepsis, 4-HPA may have a potential antioxidtive effect by cytochrome P450 2E1 to elevated the expression of HIF-1 pathways.

B. Some researches showed out that 4-HPA is one of intermediate step of chlorotyrosine, it can be catalyzed the hydroxylation of 4-HPA to 3,4-dihydroxyphenylacetic acid by 4-hydroxyphenylacetate3-hydroxylase (HPAH) which is one of mono-oxygenases found in various bacteria; and it could be tested in urine to quantify leukocyte-mediated damage in diseased tissues.

C. Because of the disorders of bidirectional communication between the brain and the gut, there are two hypotheses: one is that the gut-brain axis could offer the chances of bacteria to translocate across the intestinal mucosa and act on immune cells and neuronal cells, and it could lead to behavior changes. Two is that 4-HPA can be absorbed through gut-brain axis and the other metabolites of 4-HPA such as dopamine (DA) could be the participants of the factors of behavior changes.

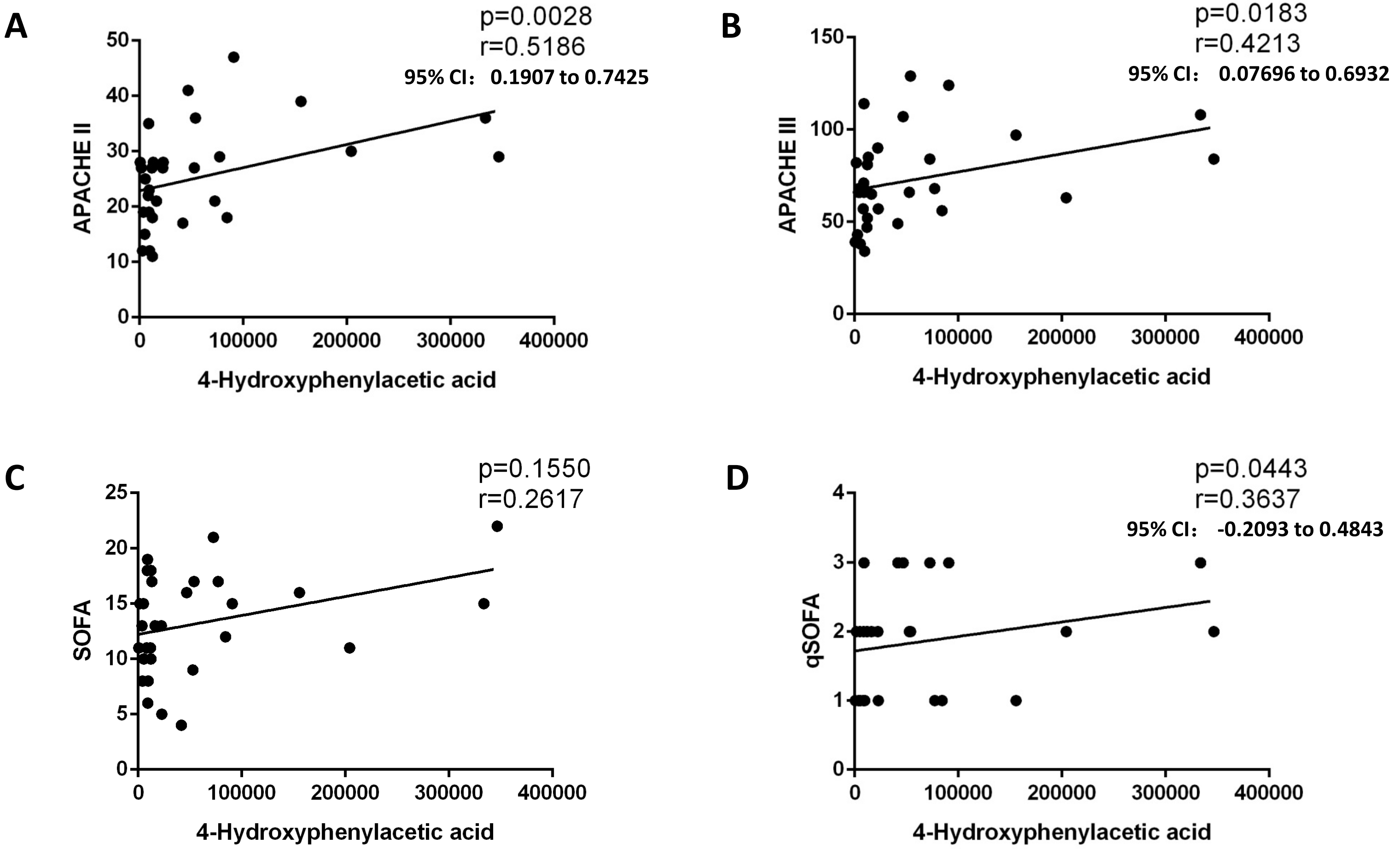

Supplement 1

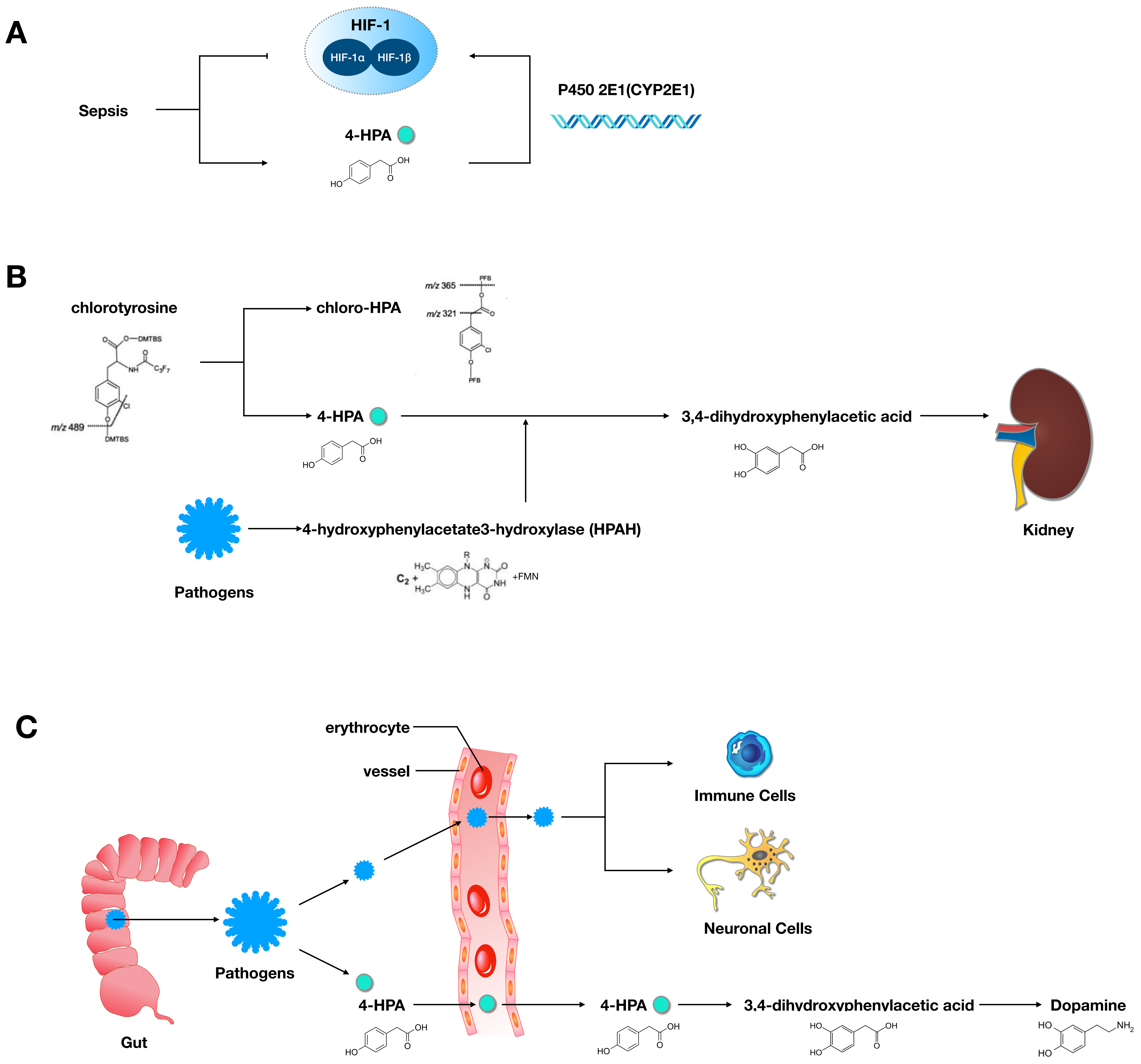
A
HIF-1
HIF-1α
HIF-1β
Sepsis
4-HPA
P450 2E1(CYP2E1)
B
chlorotyrosine
m/z 489
chloro-HPA
m/z 365
m/z 321
4-HPA
3,4-dihydroxyphenylacetic acid
Kidney
Pathogens
4-hydroxyphenylacetate3-hydroxylase (HPAH)
+FMN
C
erythrocyte
vessel
Immune Cells
Neuronal Cells
Gut
Pathogens
4-HPA
4-HPA
3,4-dihydroxyphenylacetic acid
Dopamine
